# Associations of Vaginal Pressure and Transversus Abdominis Muscle Thickness with Overactive Bladder in Women: A Cross-Sectional Study

**DOI:** 10.3390/medicina62081568

**Published:** 2026-08-16

**Authors:** Tomohiro Matsuo, Junko Tajima, Asato Otsubo, Shota Kakita, Kensuke Mitsunari, Kojiro Ohba, Ryoichi Imamura

**Affiliations:** 1 Department of Urology, Graduate School of Biomedical Sciences, Nagasaki University, Nagasaki 852-8501, Japan; tubo0316@nagasaki-u.ac.jp (A.O.); s-kakita@nagasaki-u.ac.jp (S.K.); kmitsunari@nagasaki-u.ac.jp (K.M.); ohba-k@nagasaki-u.ac.jp (K.O.); ryo-imamura@nagasaki-u.ac.jp (R.I.); 2 Division of Nursing, Nagasaki University Hospital, Nagasaki 852-8501, Japan; jtajima@nagasaki-u.ac.jp

**Keywords:** overactive bladder, transversus abdominis muscle, vaginal pressure, pelvic floor muscle contraction, lower urinary tract symptoms

## Abstract

*Background and Objectives:* Overactive bladder (OAB) has been associated with pelvic floor dysfunction, but the associations of vaginal pressure (VP) and transversus abdominis (TVA) thickness with OAB remain unclear. We examined these associations in women with lower urinary tract symptoms. *Materials and Methods:* This retrospective cross-sectional study included 213 women; 106 met the prespecified Overactive Bladder Symptom Score criteria and 107 did not. Maximal VP during voluntary pelvic floor contraction and ultrasonographic TVA thickness at rest, during maximal inspiration and maximal expiration, and during the abdominal drawing-in maneuver were compared between groups. Multivariable logistic regression adjusted for age, body mass index, number of deliveries, postmenopausal status, and hypertension. *Results:* Maximal VP and TVA thickness during the abdominal drawing-in maneuver were lower in the OAB group. In the continuous-variable model, higher VP (per 5-cmH_2_O increase: OR = 0.704, 95% CI 0.576–0.845) and greater TVA thickness during the abdominal drawing-in maneuver (per 1-mm increase: OR = 0.500, 95% CI 0.343–0.708) were associated with lower odds of OAB. Neither measure showed a consistent stepwise relationship with OAB severity. Exploratory ROC analyses yielded moderate apparent AUCs, but the cohort-derived cutoffs require external validation and should not inform clinical decisions. *Conclusions:* Lower VP and lower TVA thickness during the abdominal drawing-in maneuver were associated with OAB in this selected cohort. Concurrent cross-sectional assessment cannot establish causality or temporal relationships and cannot exclude reverse causation. Prospective studies are required before considering predictive or preventive roles, and cohort-derived cutoffs require external validation before clinical use.

## 1. Introduction

Overactive bladder (OAB) is prevalent among women, particularly with increasing age, and can substantially impair the quality of life [1]. OAB has a multifactorial etiology, and pelvic floor abnormalities have been discussed in relation to lower urinary tract function and OAB [2,3,4,5]. Pelvic floor muscle training (PFMT) is included among non-invasive treatment options for OAB [6].

Vaginal pressure (VP), measured using perineometry, quantifies the pressure increase generated during voluntary pelvic floor muscle contraction and provides an indirect measure of pelvic floor contractile performance [7,8]. The transversus abdominis (TVA), the deepest abdominal muscle, contributes to lumbopelvic stability and has been reported to show coordinated activity with the pelvic floor muscles during voluntary pelvic floor and abdominal maneuvers [9].

Pelvic floor muscle function and pelvic floor–abdominal muscle relationships have been investigated in female populations [9,10]; however, the associations of VP and TVA thickness with OAB remain insufficiently understood.

Accordingly, this exploratory cross-sectional study aimed to evaluate the associations of VP and TVA thickness with the presence and severity of OAB in women. Because VP and TVA thickness were measured during separate tasks, the present study did not directly evaluate pelvic floor–TVA coordination or coactivation. We hypothesized that lower VP and lower TVA thickness during the abdominal drawing-in maneuver would be associated with the presence of OAB.

## 2. Materials and Methods

### 2.1. Study Design and Participants

**Figure 1:**
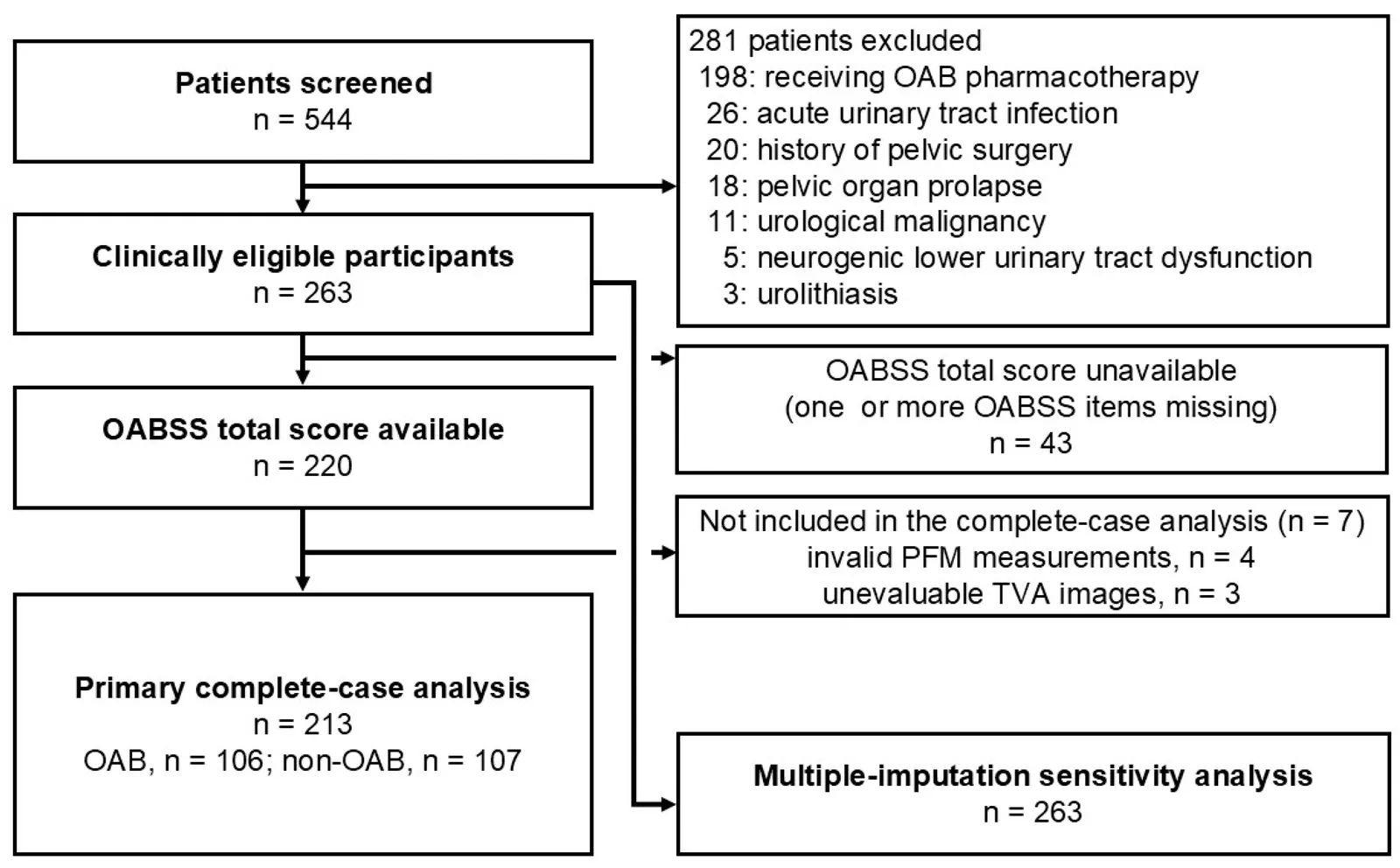
Flowchart of participant selection for the primary complete-case analysis and multiple-imputation sensitivity analysis. OAB, overactive bladder; OABSS, Overactive Bladder Symptom Score; PFM, pelvic floor muscle; TVA, transversus abdominis muscle.

This retrospective cross-sectional study included female patients aged ≥40 years who presented with at least one lower urinary tract symptom (LUTS) at Nagasaki University Hospital between January 2017 and March 2023. The age threshold was prespecified to focus on middle-aged and older women, in whom OAB is more prevalent; patients receiving OAB pharmacotherapy were excluded because treatment could modify overactive bladder symptom score (OABSS)-based group classification. A total of 544 consecutive patients were screened for eligibility. During the initial eligibility assessment, 281 patients were excluded according to the prespecified clinical criteria: 198 were already receiving drug therapy for OAB, 26 had an acute urinary tract infection, 20 had a history of pelvic surgery, 18 had pelvic organ prolapse, 11 had a urological malignancy, five had neurogenic lower urinary tract dysfunction, and three had urolithiasis. This left 263 clinically eligible participants for further assessment. Of these, 220 had complete data for all four OABSS items and therefore had a calculable OABSS total score, whereas 43 had one or more missing OABSS items. Among the 220 participants with an available OABSS total score, four lacked valid PFM measurements and three lacked evaluable TVA images. Consequently, 213 participants were included in the primary complete-case analysis. All 263 clinically eligible participants were included in the multiple-imputation sensitivity analysis after imputation of missing data (Figure 1). Childbirth history, number of deliveries, and menopausal status were retrospectively obtained from medical records.

The participants were categorized into OAB and non-OAB groups according to the prespecified OABSS criteria. VP and TVA thickness were evaluated to explore their associations with OAB. OAB was defined as an OABSS total score ≥3 with a score ≥2 on Question 3 (urgency), in accordance with the Japanese clinical guidelines for OAB [11,12]. The OABSS is a validated symptom assessment instrument, and this widely used symptom-based definition was applied for participant classification in the present study. Participants who did not meet this definition were classified as the non-OAB group. Participants in the primary complete-case cohort who met the OAB definition were further categorized according to total OABSS as having mild OAB (3–5) or moderate/severe OAB (6–15). LUTS and pelvic floor and abdominal muscle parameters, including VP and TVA thickness, were compared among the non-OAB, mild OAB, and moderate/severe OAB groups.

This study was approved by the Ethics Committee of Nagasaki University Hospital on 1 September 2018 under approval No. 20122138. The approved protocol permitted the retrospective use of clinical data collected from January 2017 onward. The study was conducted in accordance with the principles of the Declaration of Helsinki. Written informed consent was obtained from all participants.

### 2.2. Evaluation of Urinary Function

OAB symptoms were assessed using the OABSS. Objective urinary function was evaluated using voided volume (VV) and maximum urinary flow rate (Qmax), both obtained by uroflowmetry (AQUARIUS^®^ CTS; EDAP TMS Japan, Tokyo, Japan), and postvoid residual urine volume (PVR), measured by ultrasonography (Hi VISION Avius; Hitachi Medical Corporation, Tokyo, Japan).

### 2.3. Vaginal Pressure Measurement

VP was measured using the Peritron™ 9300V manometer (Cardio Design Pty Ltd., Baulkham Hills, NSW, Australia), following established protocols [7,8]. Participants were instructed to empty their bladders before testing. Measurements were performed in the supine position with the hips and knees slightly flexed. The probe was inserted approximately 3.5 cm into the vaginal canal. Each participant performed three maximal voluntary pelvic floor muscle contractions, each held for 5 s, with 30-s rest intervals. The peak pressure generated during each valid contraction was recorded. After the three maximal voluntary PFM contractions, participants were instructed to sustain a pelvic floor muscle contraction for as long as possible. The protocol-specific duration of sustained vaginal pressure above baseline was measured from pressure onset until return to resting baseline and was not interpreted as standardized PFM endurance. During each trial, the examiner visually monitored the abdominal wall, hip adductors, and gluteal muscles. Only obvious substitution patterns—global abdominal bracing, hip adduction, gluteal contraction, breath-holding, or Valsalva-like bearing down—were discarded and repeated after rest and reinstruction. Individual abdominal muscles, including the TVA and internal oblique, and subtle TVA coactivation were not assessed; no simultaneous ultrasonography or electromyography was performed. All VP measurements were performed by the same experienced clinical nurse (J.T.) with specialized training in pelvic floor function assessment. The mean of the peak pressures obtained from the three valid contractions was used as the representative VP value. Vaginal squeeze pressure measurement using the Peritron manometer has shown acceptable inter-rater reliability, although the use of the same examiner has been recommended for research assessments [7].

### 2.4. Measurement of Transversus Abdominis Muscle Thickness

TVA thickness was measured using B-mode ultrasonography with a 7.5 MHz linear transducer (Hi VISION Avius; Hitachi Medical Corporation, Tokyo, Japan). The probe position was based on the method described by Tajiri et al. [13]. Participants lay in a supine position with their knees flexed at 90° and a pillow under the head. The probe was placed perpendicular to the skin at a point 25 mm anteromedial to the midpoint between the lower costal margin and iliac crest along the right mid-axillary line. The transducer was manually stabilized by the examiner at the standardized anatomical location and orientation using light, steady contact; no external fixation device was used. All TVA ultrasonographic assessments were performed by the same board-certified urologist (T.M.) who had more than 20 years of experience in ultrasonography, including pelvic floor imaging.

TVA thickness was measured under four conditions: at rest, during maximal inspiration, during maximal expiration, and during an abdominal drawing-in maneuver. Resting TVA thickness was measured at the end of quiet expiration to minimize respiratory variation. Images during maximal inspiration and maximal expiration were acquired at the end of each respiratory phase. During the abdominal drawing-in maneuver, participants were instructed to draw the lower abdominal wall inward without breath-holding or pelvic movement, and images were acquired when maximal TVA thickening was visually observed. This condition was used to assess task-specific TVA thickness and was not considered a measure of isolated TVA activation. Muscle thickness was measured between the superficial and deep fascial borders of the TVA. The internal and external oblique muscles were not measured, and no electromyography was performed to assess muscle-specific activation or co-contraction. Three separate images were acquired under each condition, with one thickness measurement obtained from each image. The mean of the three measurements was used as the representative value. Ultrasound depth and gain settings were initially standardized, with minor adjustments made when necessary to clearly visualize the fascial borders of the TVA. Care was taken to avoid applying excessive pressure to the probe. Using the representative mean values obtained at rest and during the abdominal drawing-in maneuver, absolute TVA thickening (ΔTVA) was calculated as TVA thickness during the abdominal drawing-in maneuver minus resting TVA thickness. TVA percentage thickness change during the abdominal drawing-in maneuver was calculated as [(TVA thickness during the abdominal drawing-in maneuver − resting TVA thickness)/resting TVA thickness] × 100.

### 2.5. Statistical Analysis

All statistical analyses were performed using JMP Pro, version 17 (SAS Institute Inc., Cary, NC, USA). The distribution of each continuous variable was assessed within the relevant comparison groups using the Shapiro–Wilk test. Because every continuous variable reported in Table 1, Table 2, Table 3 and Table 4 was non-normally distributed in at least one relevant group, continuous variables are presented as medians with interquartile ranges (IQRs). Two-group comparisons were performed using the Mann–Whitney U test. Categorical variables were compared using Pearson’s chi-squared test or Fisher’s exact test, as appropriate. Comparisons of continuous variables among the non-OAB, mild OAB, and moderate/severe OAB groups were performed using the Kruskal–Wallis test. For categorical variables in the three-group analysis, Pearson’s chi-squared test or the Fisher–Freeman–Halton exact test was used, as appropriate. To account for multiple testing, the 25 omnibus *p* values reported in Table 3 and Table 4 were adjusted together using the Benjamini–Hochberg false discovery rate (FDR) procedure. Statistical significance was determined using the FDR-adjusted omnibus *p* values. When the FDR-adjusted omnibus *p* value was <0.05, Dunn’s pairwise test with Bonferroni adjustment was used for continuous variables, and pairwise categorical comparisons were Bonferroni-adjusted. Absolute TVA thickening and TVA percentage thickness change from rest to the abdominal drawing-in maneuver were compared between participants with and without OAB and among the three OAB severity groups.

Receiver operating characteristic (ROC) curves were generated for the duration of sustained PFM contraction, maximal PFM contraction pressure, and TVA thickness at rest, during maximal inspiration, during maximal expiration, and during the abdominal drawing-in maneuver to evaluate their ability to discriminate between participants with and without OAB. The optimal cutoff value for each variable was determined using the Youden index. The ROC-derived cutoff for maximal PFM contraction pressure was 20.0 cmH_2_O; values < 20.0 cmH_2_O were classified as lower VP in the cutoff-based analyses. To avoid information loss and dependence on data-derived cutoff values, maximal PFM contraction pressure and TVA thickness during the abdominal drawing-in maneuver were entered as continuous variables on their original measurement scales in the primary multivariable model. Adjusted odds ratios (ORs) and 95% confidence intervals (CIs) were expressed per 5-cmH_2_O increase in maximal PFM contraction pressure and per 1-mm increase in TVA thickness. Thus, ORs < 1 indicate lower odds of OAB with higher values of these measurements. Age, BMI, number of deliveries, and postmenopausal status were included as clinically relevant covariates regardless of their significance in univariable analysis. Hypertension was also included because it differed significantly between participants with and without OAB. History of childbirth was not included simultaneously with the number of deliveries because of conceptual overlap and potential multicollinearity.

For the secondary exploratory analyses, univariable logistic regression analyses were performed for age, BMI, history of childbirth, number of deliveries, postmenopausal status, hypertension, diabetes mellitus, hyperlipidemia, chronic kidney disease, VP < 20.0 cmH_2_O, and TVA thickness during the abdominal drawing-in maneuver <4.45 mm. Unadjusted ORs and 95% CIs were calculated for the univariable analyses. In the secondary exploratory multivariable analysis, maximal PFM contraction pressure and TVA thickness during the abdominal drawing-in maneuver were dichotomized using their respective ROC-derived cutoff values and entered into the model as VP < 20.0 cmH_2_O and TVA thickness <4.45 mm. The same covariates used in the primary model were included in this secondary model. Because these cutoffs were derived and evaluated in the same cohort, the cutoff-based model was regarded as exploratory rather than as a validation analysis and is presented only in Supplementary Table S1. The derived cutoffs should not be interpreted as validated clinical or diagnostic thresholds, and the resulting association estimates may be optimistic. Within-session, within-examiner repeatability across the three ultrasound thickness measurements obtained under each condition was evaluated using a two-way mixed-effects model with an absolute-agreement definition. Single-measure ICCs [ICC(A,1)] and average-measure ICCs for the mean of three measurements [ICC(A,3)] were calculated with 95% confidence intervals. The primary analysis was based on complete cases. The extent and patterns of missing data were summarized among all 263 clinically eligible participants, and participant characteristics were compared according to the availability of the OABSS total score. As a sensitivity analysis, missing data were handled using multiple imputation under a missing-at-random assumption. Fifty imputed datasets were generated. Missing OABSS item scores (Q1–Q4) were imputed using ordinal logistic regression, whereas missing maximal PFM contraction pressure and TVA thickness during the abdominal drawing-in maneuver were imputed using predictive mean matching. In each imputed dataset, the total OABSS was calculated from the four imputed item scores, and OAB status was passively derived according to the prespecified definition of a total OABSS ≥ 3 with an urgency score (Q3) ≥ 2. Thus, consistency among the imputed OABSS items, total OABSS, and OAB status was maintained within each imputed dataset. The imputation model included the four OABSS items, all variables included in the primary multivariable model, and the available auxiliary clinical variables. The primary multivariable logistic regression model was fitted separately in each imputed dataset, and the resulting estimates and standard errors were combined using Rubin’s rules. Statistical significance was defined as a two-sided *p* < 0.05.

Given the observational cross-sectional design, the sample size was determined by the number of consecutive eligible participants available during the study period, and no formal a priori sample size calculation was performed. Rather than calculating observed post-hoc power, which provides limited information beyond the observed effect estimate and *p* value, we performed a post-hoc sensitivity analysis to estimate the minimum detectable standardized between-group difference, assuming a two-sided two-sample *t* test. With 106 participants with OAB and 107 participants without OAB, an α level of 0.05, and 80% power, the minimum detectable standardized between-group difference was approximately 0.39.

## 3. Results

### 3.1. Patient Characteristics and Differences in Urological Parameters Between OAB and Non-OAB Groups

Of the 263 clinically eligible participants, 220 had an available OABSS total score. After excluding four participants without valid PFM measurements and three without evaluable TVA images, 213 participants were included in the primary complete-case analysis. Table 1 summarizes the demographic and clinical characteristics of these 213 participants: 106 (49.8%) were assigned to the OAB group and 107 (50.2%) to the non-OAB group. The rates of childbirth history and postmenopausal status, as well as the number of deliveries, did not differ significantly between the OAB and non-OAB groups. Participants in the OAB group were significantly older than those in the non-OAB group (71.0 [64.0–76.8] vs. 63.0 [56.0–72.0] years, *p* < 0.001) and had a higher BMI (24.1 [21.2–26.7] vs. 21.5 [20.1–24.3] kg/m^2^, *p* < 0.001). The prevalence of hypertension was also significantly higher in the OAB group than in the non-OAB group (61.3% vs. 31.8%, *p* < 0.001). However, the rates of diabetes mellitus, chronic kidney disease, and hyperlipidemia did not significantly differ between the two groups. No material differences were observed in the measured demographic and clinical characteristics between participants with available and unavailable OABSS total scores. The largest absolute standardized difference was 0.12 for postvoid residual urine volume (Supplementary Table S3).

The OAB group had significantly higher scores across all items of the OABSS, particularly for urgency (Q3: 2.0 [2.0–4.0] vs. 0.0 [0.0–1.0], *p* < 0.001) and urgency incontinence (Q4: 2.0 [1.0–3.0] vs. 0.0 [0.0–0.0], *p* < 0.001). Objective urinary assessments revealed that the OAB group had significantly lower voided volumes (174.0 [145.0–243.0] vs. 245.0 [174.0–297.0] mL, *p* < 0.001), reduced maximum flow rates (19.7 [18.4–24.3] vs. 24.3 [18.4–28.6] mL/s, *p* = 0.003), and higher postvoid residual urine volumes (35.5 [23.2–54.0] vs. 23.0 [12.0–43.0] mL, *p* < 0.001) compared to the non-OAB group.

### 3.2. Differences in VP and TVA Thickness Between OAB and Non-OAB Groups

The duration of sustained PFM contraction did not differ significantly between the OAB and non-OAB groups (11.0 [6.0–16.0] vs. 13.0 [6.0–15.0] s, *p* = 0.965), whereas maximal PFM contraction pressure was significantly lower in the OAB group (15.0 [13.0–21.0] vs. 25.0 [21.0–34.5] cmH_2_O, *p* < 0.001) (Table 2).

The OAB group had lower TVA thickness at rest (2.4 [2.0–2.9] vs. 2.5 [2.1–3.1] mm, *p* = 0.014), during maximal inspiration (2.1 [2.0–2.4] vs. 2.4 [2.0–2.9] mm, *p* < 0.001), during maximal expiration (3.5 [3.0–3.9] vs. 4.0 [3.6–4.6] mm, *p* < 0.001), and during the abdominal drawing-in maneuver (4.2 [3.4–5.1] vs. 5.2 [4.3–6.1] mm, *p* < 0.001). Absolute TVA thickening and TVA percentage thickness change were also lower in the OAB group (1.9 [1.1–2.5] vs. 2.5 [1.5–3.6] mm, *p* < 0.001, and 82.5 [52.3–103.3]% vs. 89.1 [48.4–149.0]%, *p* = 0.016, respectively) (Table 2). Within-session, within-examiner repeatability was good to excellent for single measurements and excellent for the mean of three measurements. ICC(A,1) values ranged from 0.86 to 0.91, whereas ICC(A,3) values ranged from 0.95 to 0.97 across the four measurement conditions (Supplementary Table S2).

### 3.3. Patient Characteristics, VP, and TVA Thickness According to OAB Severity

Age differed significantly across the OAB severity groups (non-OAB: 63.0 [56.0–72.0]; mild OAB: 68.0 [58.0–77.0]; moderate/severe OAB: 71.0 [66.0–76.0] years; FDR-adjusted *p* < 0.001) (Table 3). BMI also differed across the groups (21.5 [20.1–24.3], 25.1 [20.1–28.4], and 24.1 [22.1–25.5] kg/m^2^, respectively; FDR-adjusted *p* = 0.003). The prevalence of hypertension differed across the groups (FDR-adjusted *p* < 0.001), whereas diabetes mellitus, chronic kidney disease, and hyperlipidemia did not.

Voided volume was lower in both OAB groups than in the non-OAB group (non-OAB: 245.0 [174.0–297.0]; mild OAB: 197.0 [145.0–244.0]; moderate/severe OAB: 173.0 [142.0–243.0] mL; FDR-adjusted *p* < 0.001). Postvoid residual urine volume was higher in the moderate/severe OAB group than in both the non-OAB and mild OAB groups (43.0 [32.0–57.0] vs. 23.0 [12.0–43.0] and 24.0 [12.0–36.0] mL, respectively; FDR-adjusted *p* < 0.001). Maximum flow rate was lower in the moderate/severe OAB group than in the non-OAB group (19.2 [17.5–22.3] vs. 24.3 [18.4–28.6] mL/s), but the other pairwise comparisons were not significant after adjustment (FDR-adjusted omnibus *p* = 0.003) (Table 3).

Maximal PFM contraction pressure and TVA thickness differed significantly among the three groups, with FDR-adjusted omnibus *p* values < 0.001 for maximal PFM contraction pressure and for TVA thickness under all four measurement conditions (Table 4). Absolute TVA thickening was lower in both OAB groups than in the non-OAB group (mild OAB: 1.5 [1.1–2.1]; moderate/severe OAB: 2.0 [1.1–2.5]; non-OAB: 2.5 [1.5–3.6] mm; FDR-adjusted *p* < 0.001). TVA percentage thickness change showed a nominal difference in the unadjusted analysis (*p* = 0.041), but this did not remain significant after FDR correction (FDR-adjusted *p* = 0.058). Overall, neither maximal PFM contraction pressure nor the TVA measurements showed a consistent stepwise relationship with OAB severity.

### 3.4. Exploratory ROC Analyses of VP and TVA Thickness for OAB Status

As shown in Table 5, exploratory ROC analyses were performed to assess the apparent discrimination of PFM and TVA parameters for OAB status. Maximal VP during PFM contraction showed moderate apparent discrimination, with an AUC of 0.756 (sensitivity = 0.698, specificity = 0.776, *p* < 0.001) and a ROC-derived cutoff of 20.0 cmH_2_O. Similarly, TVA thickness during the abdominal drawing-in maneuver showed moderate apparent discrimination, with an AUC of 0.730 (sensitivity = 0.660, specificity = 0.692, *p* < 0.001) and a ROC-derived cutoff of 4.45 mm.

### 3.5. Factors Associated with OAB in the Primary Continuous-Variable Model

The primary multivariable analysis included all 213 participants in the complete-case cohort. In the primary continuous-variable model, hypertension (OR = 4.891, 95% CI 2.330–10.268, *p* < 0.001) was associated with higher odds of OAB, whereas higher maximal PFM contraction pressure (per 5-cmH_2_O increase: OR = 0.704, 95% CI 0.576–0.845, *p* < 0.001) and greater TVA thickness during the abdominal drawing-in maneuver (per 1-mm increase: OR = 0.500, 95% CI 0.343–0.708, *p* < 0.001) were associated with lower odds of OAB after multivariable adjustment (Table 6). A secondary cutoff-based analysis is presented in Supplementary Table S1 solely for exploratory purposes. Because the cutoffs were derived and evaluated in the same cohort, the resulting association estimates may be optimistic, and the cutoffs should not be interpreted as validated clinical thresholds. In the multiple-imputation sensitivity analysis of all 263 clinically eligible participants, hypertension (adjusted OR = 4.523, 95% CI 2.297–8.906, *p* < 0.001) was associated with higher odds of OAB, whereas higher maximal PFM contraction pressure (per 5-cmH_2_O increase: adjusted OR = 0.716, 95% CI 0.603–0.851, *p* < 0.001) and greater TVA thickness during the abdominal drawing-in maneuver (per 1-mm increase: adjusted OR = 0.526, 95% CI 0.382–0.724, *p* < 0.001) were associated with lower odds of OAB. These results were consistent with those of the primary complete-case analysis (Supplementary Table S4). In the post-hoc sensitivity analysis, the minimum standardized between-group difference detectable with 80% power at a two-sided α level of 0.05 was approximately 0.39. Thus, smaller standardized between-group differences may not have been reliably detected.

## 4. Discussion

This study examined VP as an indirect measure of pelvic floor contractile performance and task-specific ultrasonographic TVA thickness in women with and without OAB. Maximal PFM contraction pressure and TVA thickness during the abdominal drawing-in maneuver were significantly lower in women with OAB than in those without OAB. In the primary continuous-variable model, higher maximal PFM contraction pressure and greater TVA thickness during the abdominal drawing-in maneuver were associated with lower odds of OAB after adjustment for clinically relevant covariates. However, neither measure showed a consistent stepwise relationship with OAB severity. Because VP and TVA thickness were measured during separate tasks, these findings do not demonstrate impaired PFM–TVA coordination or coactivation. VP should be interpreted as an indirect measure of pelvic floor contractile performance, whereas ultrasonographic TVA thickness and task-related thickness changes are morphological measurements obtained under standardized task conditions and do not directly quantify muscle activation, strength, contractile performance, or neuromuscular function. These findings provide additional observational evidence regarding the musculoskeletal characteristics associated with OAB but do not establish causality.

OAB is a multifactorial symptom syndrome [14], and pelvic floor abnormalities have been discussed in relation to lower urinary tract function [2,3,4,5]. In our cohort, maximal PFM contraction pressure was lower in women with OAB and remained associated with OAB after adjustment. This supports an association between pelvic floor contractile performance and OAB but does not establish its direction, mechanism, or therapeutic implications. Although PFMT is established for urinary incontinence [15,16] and has been investigated in OAB [6], the present study did not assess treatment efficacy.

TVA thickness was lower in the OAB group under all measurement conditions. Absolute TVA thickening and percentage thickness change were also lower, indicating differences in both task-specific thickness and task-related thickness change. Because these two change measures were evaluated only in group comparisons and were not included in the primary multivariable model, they are supportive exploratory findings rather than independent factors associated with OAB. Lower TVA thickness during the abdominal drawing-in maneuver remained associated with OAB after adjustment for the measured covariates.

Previous studies have examined pelvic floor–abdominal and pelvic floor–diaphragm relationships, as well as combined training approaches [17,18,19,20,21,22,23]. In the present study, TVA thickness values during maximal expiration and the abdominal drawing-in maneuver were slightly higher in the moderate/severe OAB group than in the mild OAB group, although both OAB groups had lower values than the non-OAB group. This non-monotonic pattern does not support a dose–response relationship. Given the multifactorial nature of OABSS, TVA thickness should not be interpreted as a direct marker of symptom severity or PFM–TVA coordination.

Hypertension remained associated with OAB after multivariable adjustment. However, the mechanisms underlying this association were not assessed, and residual confounding cannot be excluded. Although age was significantly associated with OAB in the univariable analysis, its estimate was attenuated and was not statistically significant after multivariable adjustment. BMI was also not statistically significant in the primary continuous-variable model (adjusted OR = 1.102, 95% CI 0.989–1.232, *p* = 0.080). These non-significant findings should not be interpreted as evidence of no association, because modest associations may not have been detected with the available sample size. Although age and BMI were included a priori in the primary multivariable model, statistical adjustment may reduce but cannot eliminate confounding arising from these baseline differences, particularly in the presence of possible nonlinear relationships, measurement error, and unmeasured age- or body-habitus-related factors. The cross-sectional design also precludes formal assessment of mediation or causal ordering among these variables and OAB.

VP and TVA ultrasonography provide objective measures of pelvic floor contractile performance and task-specific TVA morphology, respectively [7,8,13]. Exploratory ROC analyses showed moderate apparent discrimination for maximal PFM contraction pressure and TVA thickness during the abdominal drawing-in maneuver; however, their incremental value beyond symptom-based assessment was not evaluated.

Despite these findings, this study had several limitations. First, VP, TVA thickness, and OAB status were assessed contemporaneously in this retrospective cross-sectional study, precluding determination of temporal relationships or causality. Reverse causation cannot be excluded: lower VP or TVA thickness may precede OAB, whereas urgency-related behavioral adaptations or chronic bladder dysfunction may also affect these measurements. Accordingly, the observed findings should be interpreted only as contemporaneous associations and do not establish predictive or preventive roles. Prospective longitudinal studies are required to determine temporal ordering and evaluate whether either measure has predictive or preventive utility. Moreover, the retrospective use of clinical data collected between January 2017 and March 2023 limited prospective control over the completeness and uniformity of some clinical variables and may have introduced information bias.

Residual confounding may also remain despite multivariable adjustment. In addition, the single-center setting, restriction to women aged ≥40 years, and other eligibility criteria limit generalizability. Excluding 198 patients receiving OAB treatment was intended to reduce treatment-related misclassification but may have preferentially removed women with clinically recognized OAB, thereby altering cohort composition. Because excluded-patient data were incomplete, the direction and magnitude of this selection bias could not be assessed. In addition, OABSS total scores were unavailable for 43 of the 263 clinically eligible participants because one or more OABSS items were missing. This may have introduced selection bias if questionnaire completion was associated with unmeasured symptom severity or other participant characteristics. Although the multiple-imputation sensitivity analysis yielded results consistent with those of the primary complete-case analysis, multiple imputation relies on the missing-at-random assumption. Therefore, bias arising from a missing-not-at-random mechanism cannot be excluded. No formal a priori sample size calculation was performed because the sample comprised consecutive eligible participants during the study period. The post-hoc sensitivity analysis indicated that, with 106 participants with OAB and 107 without OAB, the minimum standardized between-group difference detectable with 80% power at a two-sided α level of 0.05 was approximately 0.39. Therefore, the study may have been insufficiently powered to detect smaller but clinically meaningful between-group differences. Accordingly, non-significant findings, particularly those accompanied by wide confidence intervals, should not be interpreted as evidence of no association. This limitation is particularly relevant to age and postmenopausal status in the multivariable analysis.

Second, VP and TVA thickness were assessed using standardized protocols; however, some variability related to examiner technique and participant cooperation may still exist. All ultrasound assessments were conducted by a single experienced urologist (T.M.), and all VP measurements were performed by a single trained clinical nurse (J.T.) to minimize this variability. Formal blinding to OABSS results and pretest confirmation by digital palpation were not documented. Although four participants were excluded because valid VP measurements could not be obtained and three were excluded because TVA images were missing or had indistinct fascial borders, trial-level counts of repeated or discarded VP contractions and reacquired or discarded ultrasound images were unavailable. Study-specific calibration records for the VP measurement device and ultrasound system were unavailable for retrospective verification. In addition, BMI may influence ultrasound-based assessment of TVA thickness through subcutaneous fat attenuation and image quality. Although ultrasound depth and gain settings were standardized as much as possible, measurement bias related to body habitus cannot be fully excluded. Although the TVA layer was anatomically delineated using its superficial and deep fascial borders, the abdominal drawing-in maneuver is not specific to the TVA and may involve co-contraction of the internal and external oblique muscles and other components of the abdominal core. Therefore, the measured thickness represents task-specific TVA morphology obtained during a broader abdominal-core activation pattern and should not be interpreted as isolated TVA activation or function. Although within-session, within-examiner repeatability was good to excellent for single measurements and excellent for the mean of three measurements, inter-rater reliability and between-session reproducibility could not be evaluated because all measurements were performed by a single examiner within the same session. Future studies should assess inter-rater and between-session reliability. VP and TVA thickness were measured during separate tasks; therefore, simultaneous PFM–TVA coordination or coactivation could not be evaluated. Future studies using simultaneous PFM electromyography and TVA ultrasonography are needed to assess this relationship directly.

Third, although we focused on the TVA as a representative abdominal core muscle, other components of the abdominal and spinal musculature, such as the internal oblique, multifidus, and diaphragm, may also play important roles in bladder control and postural regulation. Expanding assessments to include these additional muscles would provide a more comprehensive characterization of musculoskeletal factors associated with OAB.

Fourth, although patients receiving pharmacotherapy for OAB were excluded, information on diuretics, antihypertensive agents, anticholinergic medications used for non-OAB indications, and other drugs that could affect lower urinary tract function was not systematically available. Because these data were unavailable, medication-related residual confounding could not be addressed in a sensitivity analysis and may have affected the adjusted associations.

Fifth, although childbirth history, number of deliveries, and menopausal status were available and evaluated, detailed obstetric and hormonal information, including mode of delivery, operative vaginal delivery, childbirth-related pelvic floor trauma, and hormone therapy, was not systematically recorded. The number of deliveries was modeled as a continuous variable, and possible nonlinear or threshold associations were not examined. Therefore, residual confounding related to reproductive and hormonal factors cannot be completely excluded. In addition, childbirth history and postmenopausal status were highly prevalent in this cohort, resulting in limited precision and relatively wide confidence intervals. In particular, the wide confidence interval for postmenopausal status was compatible with associations in either direction; therefore, its non-significant estimate should not be interpreted as evidence of no association.

Sixth, the cutoff values for VP and TVA thickness were derived and evaluated in the same cohort without internal or external validation. Accordingly, both their apparent discriminative performance and the association estimates obtained from the cutoff-based model may be affected by optimism. The cutoff-based analyses should therefore be considered exploratory and cannot establish clinically applicable thresholds. External validation in an independent cohort is required before any clinical application of these thresholds.

Seventh, information on constipation, symptom duration, physical activity, and psychological stress was not systematically available. Residual confounding from these unmeasured clinical and behavioral factors therefore cannot be excluded.

Accordingly, these measures should not be interpreted as predictive or preventive markers and should not guide diagnosis or treatment selection. Evidence for PFMT in women with OAB remains heterogeneous [6], and our findings do not establish that pelvic floor or core training improves OAB symptoms or that VP and TVA thickness are treatment outcome measures. Spinal stabilization exercises focusing on the pelvic floor have shown potential benefits in women with OAB [24], but further studies are needed to establish the clinical relevance of pelvic floor and abdominal core rehabilitation.

## 5. Conclusions

This retrospective cross-sectional study found that lower maximal PFM contraction pressure, assessed using vaginal pressure, and lower TVA thickness during the abdominal drawing-in maneuver were associated with OAB after multivariable adjustment. Neither measure showed a consistent stepwise relationship with OAB severity. Because VP, TVA thickness, and OAB status were assessed contemporaneously, temporal relationships and causality cannot be determined, and reverse causation cannot be excluded. Accordingly, these findings do not establish underlying mechanisms, incremental clinical utility, or predictive or preventive roles for either measure. Prospective longitudinal studies are required before such roles can be considered. The cohort-derived cutoffs remain exploratory and require external validation before any clinical application.

## Figures and Tables

**Table 1:** Participant characteristics and differences in urological parameters between the OAB and non-OAB groups.

	Overall	Non-OAB	OAB	*p*-Value
Number of patients, *n* (%)	213	107 (50.2)	106 (49.8)	-
Age (years)	67.0 [59.0–74.0]	63.0 [56.0–72.0]	71.0 [64.0–76.8]	<0.001
BMI (kg/m^2^)	22.8 [20.6–25.2]	21.5 [20.1–24.3]	24.1 [21.2–26.7]	<0.001
Female pelvic floor-related factors				
History of childbirth, *n* (%)	204 (95.8)	101 (94.4)	103 (97.2)	0.498
Number of deliveries	2.0 [2.0–3.0]	2.0 [1.5–3.0]	2.0 [2.0–3.0]	0.106
Postmenopausal status, *n* (%)	196 (92.0)	98 (91.6)	98 (92.5)	0.816
Comorbidity				
Hypertension, *n* (%)	99 (46.5)	34 (31.8)	65 (61.3)	<0.001
Diabetes mellitus, *n* (%)	57 (26.8)	26 (24.3)	31 (29.2)	0.415
Chronic kidney disease, *n* (%)	57 (26.8)	23 (21.5)	34 (32.1)	0.081
Hyperlipidemia, *n* (%)	57 (26.8)	31 (29.0)	26 (24.5)	0.464
Urological parameters				
OABSS				
Q1 daytime frequency	1.0 [0.0–1.0]	0.0 [0.0–1.0]	1.0 [1.0–1.0]	<0.001
Q2 nighttime frequency	1.0 [1.0–2.0]	1.0 [0.0–2.0]	2.0 [1.0–2.0]	<0.001
Q3 urgency	2.0 [0.0–2.0]	0.0 [0.0–1.0]	2.0 [2.0–4.0]	<0.001
Q4 urgency incontinence	0.0 [0.0–2.0]	0.0 [0.0–0.0]	2.0 [1.0–3.0]	<0.001
Total OABSS	4.0 [2.0–6.0]	2.0 [1.0–3.0]	6.0 [5.0–9.0]	<0.001
Objective findings				
VV (mL)	198.0 [157.0–274.0]	245.0 [174.0–297.0]	174.0 [145.0–243.0]	<0.001
Qmax (mL/s)	21.2 [18.4–26.3]	24.3 [18.4–28.6]	19.7 [18.4–24.3]	0.003
PVR (mL)	31.0 [16.0–46.0]	23.0 [12.0–43.0]	35.5 [23.2–54.0]	<0.001

Continuous variables are presented as medians [interquartile ranges] and were compared using the Mann–Whitney U test. Categorical variables are presented as numbers (%) and were compared using Pearson’s chi-squared test or Fisher’s exact test, as appropriate. OAB, overactive bladder; BMI, body mass index; OABSS, overactive bladder symptom score; VV, voided volume; Qmax, maximum flow rate; PVR, postvoid residual urine.

**Table 2:** Differences in VP and TVA thickness between the OAB and non-OAB groups.

	Overall	Non-OAB	OAB	*p*-Value
VP				
Duration of sustained PFM contraction (s)	12.0 [6.0–15.0]	13.0 [6.0–15.0]	11.0 [6.0–16.0]	0.965
Maximal PFM contraction pressure (cmH_2_O)	21.0 [14.0–32.0]	25.0 [21.0–34.5]	15.0 [13.0–21.0]	<0.001
TVA thickness				
At rest (mm)	2.5 [2.1–3.0]	2.5 [2.1–3.1]	2.4 [2.0–2.9]	0.014
During maximal inspiration (mm)	2.2 [2.0–2.5]	2.4 [2.0–2.9]	2.1 [2.0–2.4]	<0.001
During maximal expiration (mm)	3.8 [3.2–4.2]	4.0 [3.6–4.6]	3.5 [3.0–3.9]	<0.001
During the abdominal drawing-in maneuver (mm)	4.5 [3.9–5.7]	5.2 [4.3–6.1]	4.2 [3.4–5.1]	<0.001
Absolute TVA thickening (ΔTVA), mm	2.0 [1.2–2.9]	2.5 [1.5–3.6]	1.9 [1.1–2.5]	<0.001
TVA percentage thickness change, %	83.8 [48.4–122.0]	89.1 [48.4–149.0]	82.5 [52.3–103.3]	0.016

Continuous variables are presented as medians [interquartile ranges] and were compared using the Mann–Whitney U test. OAB, overactive bladder; VP, vaginal pressure; PFM, pelvic floor muscle; TVA, transversus abdominis muscle.

**Table 3:** Patient characteristics and urological parameters according to OAB severity.

	Non-OAB	Mild OAB	Moderate/Severe OAB	Unadjusted *p*-Value	FDR-Adjusted *p*-Value
Number of patients, *n* (%)	107 (50.2)	37 (17.4)	69 (32.4)	-	-
Age (years)	63.0 [56.0–72.0]	68.0 [58.0–77.0]	71.0 [66.0–76.0] **	<0.001	<0.001
BMI (kg/m^2^)	21.5 [20.1–24.3]	25.1 [20.1–28.4] *	24.1 [22.1–25.5] **	0.002	0.003
Female pelvic floor-related factors					
History of childbirth, *n* (%)	101 (94.4)	37 (100.0)	66 (95.7)	0.528	0.600
Number of deliveries	2.0 [1.5–3.0]	2.0 [2.0–2.0]	2.0 [2.0–3.0]	0.071	0.093
Postmenopausal status, *n* (%)	98 (91.6)	32 (86.5)	66 (95.7)	0.232	0.276
Comorbidity					
Hypertension, *n* (%)	34 (31.8)	20 (54.1) *	45 (65.2) **	<0.001	<0.001
Diabetes mellitus, *n* (%)	26 (24.3)	11 (29.7)	20 (29.0)	0.715	0.723
Chronic kidney disease, *n* (%)	23 (21.5)	10 (27.0)	24 (34.8)	0.151	0.189
Hyperlipidemia, *n* (%)	31 (29.0)	10 (27.0)	16 (23.2)	0.699	0.723
Urological parameters					
OABSS					
Q1 daytime frequency	0.0 [0.0–1.0]	1.0 [0.0–1.0] *	1.0 [1.0–1.0] **^, ††^	<0.001	<0.001
Q2 nighttime frequency	1.0 [0.0–2.0]	1.0 [0.0–2.0]	2.0 [1.0–3.0] **^, ††^	<0.001	<0.001
Q3 urgency	0.0 [0.0–1.0]	2.0 [2.0–2.0] **	3.0 [2.0–4.0] **^, †^	<0.001	<0.001
Q4 urgency incontinence	0.0 [0.0–0.0]	1.0 [0.0–1.0] **	2.0 [2.0–3.0] **^, ††^	<0.001	<0.001
Total OABSS	2.0 [1.0–3.0]	5.0 [4.0–5.0] **	8.0 [6.0–10.0] **^, ††^	<0.001	<0.001
Objective findings					
VV (mL)	245.0 [174.0–297.0]	197.0 [145.0–244.0] *	173.0 [142.0–243.0] **	<0.001	<0.001
Qmax (mL/s)	24.3 [18.4–28.6]	21.2 [18.5–25.4]	19.2 [17.5–22.3] **	0.002	0.003
PVR (mL)	23.0 [12.0–43.0]	24.0 [12.0–36.0]	43.0 [32.0–57.0] **^, ††^	<0.001	<0.001

Continuous variables are presented as medians [interquartile ranges], and categorical variables as numbers (%). Omnibus *p* values were calculated using the Kruskal–Wallis test for continuous variables and Pearson’s chi-squared test or the Fisher–Freeman–Halton exact test for categorical variables, as appropriate. The 17 omnibus *p* values presented in this table and the eight presented in Table 4 were adjusted jointly using the Benjamini–Hochberg FDR procedure. When the FDR-adjusted omnibus *p* value was <0.05, Dunn’s pairwise tests with Bonferroni adjustment were used for continuous variables; pairwise categorical comparisons were Bonferroni-adjusted. OAB, overactive bladder; FDR, false discovery rate; BMI, body mass index; OABSS, overactive bladder symptom score; VV, voided volume; Qmax, maximum flow rate; PVR, postvoid residual urine; * *p* < 0.05 vs. non-OAB; ** *p* < 0.01 vs. non-OAB; ^†^ *p* < 0.05 vs. mild OAB; ^††^ *p* < 0.01 vs. mild OAB.

**Table 4:** VP and TVA thickness according to OAB severity.

	Non- OAB	Mild OAB	Moderate/Severe OAB	Unadjusted *p*-Value	FDR-Adjusted *p*-Value
VP					
Duration of sustained PFM contraction (s)	13.0 [6.0–15.0]	7.0 [5.0–16.0]	13.0 [6.0–16.0]	0.723	0.723
Maximal PFM contraction pressure (cmH_2_O)	25.0 [21.0–34.5]	15.0 [13.0–21.0] **	15.0 [13.0–21.0] **	<0.001	<0.001
TVA thickness					
At rest (mm)	2.5 [2.1–3.1]	2.1 [1.5–2.5] **	2.6 [2.3–3.0] ^††^	<0.001	<0.001
During maximal inspiration (mm)	2.4 [2.0–2.9]	2.0 [1.9–2.2] **	2.2 [2.0–2.4] ^†^	<0.001	<0.001
During maximal expiration (mm)	4.0 [3.6–4.6]	3.3 [2.6–3.9] **	3.5 [3.1–3.9] **	<0.001	<0.001
During the abdominal drawing-in maneuver (mm)	5.2 [4.3–6.1]	3.5 [2.8–4.2] **	4.2 [3.5–5.2] **	<0.001	<0.001
Absolute TVA thickening (ΔTVA), mm	2.5 [1.5–3.6]	1.5 [1.1–2.1] **	2.0 [1.1–2.5] *	<0.001	<0.001
TVA percentage thickness change, %	89.1 [48.4–149.0]	86.2 [56.1–107.7]	82.5 [43.2–101.8]	0.041	0.058

Continuous variables are presented as medians [interquartile ranges]. Omnibus *p* values were calculated using the Kruskal–Wallis test. The eight omnibus *p* values presented in this table and the 17 presented in Table 3 were adjusted jointly using the Benjamini–Hochberg FDR procedure. Dunn’s pairwise tests with Bonferroni adjustment are presented only when the corresponding FDR-adjusted omnibus *p* value was <0.05. OAB, overactive bladder; FDR, false discovery rate; VP, vaginal pressure; PFM, pelvic floor muscle; TVA, transversus abdominis muscle; * *p* < 0.05 vs. non-OAB; ** *p* < 0.01 vs. non-OAB; ^†^ *p* < 0.05 vs. mild OAB; ^††^ *p* < 0.01 vs. mild OAB.

**Table 5:** Exploratory receiver operating characteristic analysis of vaginal pressure and transversus abdominis muscle thickness in relation to OAB status.

	Cutoff Value	Sensitivity	Specificity	AUC	*p*-Value
VP					
Duration of sustained PFM contraction (s)	14.0	0.443	0.626	0.502	0.571
Maximal PFM contraction pressure (cmH_2_O)	20.0	0.698	0.776	0.756	<0.001
TVA thickness					
At rest (mm)	3.05	0.887	0.308	0.597	0.002
During maximal inspiration (mm)	2.25	0.717	0.598	0.652	<0.001
During maximal expiration (mm)	3.90	0.764	0.617	0.701	<0.001
During the abdominal drawing-in maneuver (mm)	4.45	0.660	0.692	0.730	0.001

Receiver operating characteristic (ROC) analysis was performed, and optimal cutoff values were determined using the Youden index. OAB, overactive bladder; VP, vaginal pressure; TVA, transversus abdominis muscle; AUC, area under the curve; PFM, pelvic floor muscle.

**Table 6:** Primary multivariable logistic regression analysis of factors associated with OAB in the complete-case cohort (*n* = 213).

	Adjusted OR	95% CI	*p*-Value
Age, per 1-year increase	1.016	0.971–1.064	0.486
BMI, per 1-kg/m^2^ increase	1.102	0.989–1.232	0.080
Number of deliveries, per 1-delivery increase	1.095	0.747–1.613	0.642
Postmenopausal status, yes vs. no	0.416	0.088–1.969	0.267
Hypertension, yes vs. no	4.891	2.330–10.268	<0.001
Maximal PFM contraction pressure, per 5-cmH_2_O increase	0.704	0.576–0.845	<0.001
TVA thickness during the abdominal drawing-in maneuver, per 1-mm increase	0.500	0.343–0.708	<0.001

Adjusted ORs and 95% CIs were estimated using multivariable logistic regression analysis. The analysis included 213 participants with complete data for OAB status, PFM and TVA measurements, and all covariates included in the model. For maximal PFM contraction pressure and TVA thickness, ORs are reported per increase on the original measurement scales; ORs < 1 indicate lower odds of OAB with higher values. OAB, overactive bladder; OR, odds ratio; CI, confidence interval; BMI, body mass index; PFM, pelvic floor muscle; TVA, transversus abdominis muscle.

## Data Availability

The data supporting this study are available from the corresponding author upon request. The data are not publicly accessible owing to privacy and ethical considerations.

## Supplementary Materials

The following supporting information can be downloaded at: https://www.mdpi.com/article/10.3390/medicina62081568/s1, Table S1: Exploratory cutoff-based univariable and multivariable logistic regression analyses of factors associated with OAB; Table S2: Within-session, within-examiner repeatability of transversus abdominis muscle thickness measurements; Table S3: Extent and patterns of missing data among the 263 clinically eligible participants; Table S4: Sensitivity analysis using multiple imputation for factors associated with overactive bladder among the 263 clinically eligible participants.

## Abbreviations

The following abbreviations are used in this manuscript:

OAB	Overactive bladder
PFMT	Pelvic floor muscle training
VP	Vaginal pressure
TVA	Transversus abdominis muscle
IQR	Interquartile range
LUTS	Lower urinary tract symptoms
OABSS	Overactive bladder symptom score
ROC	Receiver operating characteristic
AUC	Area under the curve
ORs	Odds ratios
CIs	Confidence intervals
BMI	Body mass index
PFM	Pelvic floor muscle
FDR	False discovery rate
VV	Voided volume
Qmax	Maximum flow rate
PVR	Postvoid residual urine
