# Supplementary material for: Associations of Vaginal Pressure and Transversus Abdominis Muscle Thickness with Overactive Bladder in Women: A Cross-Sectional Study
Source: Medicina (Kaunas). 2026 Aug 16;62(8):1568. doi: 10.3390/medicina62081568 (PMC13515797; doi:10.3390/medicina62081568)
Supplement: Supplementary file 1 [file medicina-62-01568-s001.zip › medicina-4397272-supplementary.pdf]

Supplementary Table S1. Exploratory cutoff-based univariable and multivariable logistic regression analyses of factors associated with OAB.

	Univariable analysis			Multivariable analysis		
	OR	95% CI	P-value	OR	95% CI	P-value
Age, per 1-year increase	1.055	1.026–1.087	< 0.001	1.018	0.973–1.065	0.433
BMI, per 1 kg/m^2 increase	1.162	1.069–1.269	< 0.001	1.110	1.000–1.236	0.049
History of childbirth, yes vs no	2.039	0.522–9.898	0.323	–	–	–
Number of deliveries, 1-delivery increase	1.336	1.087–1.773	0.034	1.023	0.690–1.515	0.911
Postmenopausal status, yes vs no	1.125	0.413–3.111	0.816	1.723	0.365–8.131	0.492
Hypertension, yes vs no	3.404	1.995–6.039	< 0.001	4.681	2.287–9.580	< 0.001
Diabetes mellitus, yes vs no	1.288	0.702–2.378	0.415	–	–	–
Hyperlipidemia, yes vs no	0.798	0.431–1.463	0.464	–	–	–
Chronic kidney disease, yes vs no	1.724	0.936–3.221	0.081	–	–	–
VP (<20.0 cmH_2O)	8.000	4.385–15.051	< 0.001	5.693	2.690–12.049	< 0.001
TVA thickness during the abdominal drawing-in maneuver (<4.45 mm)	4.360	2.477–7.828	< 0.001	2.778	1.314–5.873	0.008

ORs and 95% CIs were estimated using univariable and multivariable logistic regression analyses.

Because the VP and TVA cutoffs were derived and evaluated in the same cohort, the cutoff-based association estimates may be optimistic. This analysis is exploratory, and the cohort-derived cutoffs should not be interpreted as validated clinical or diagnostic thresholds. OAB, overactive bladder; OR, odds ratio; CI, confidence interval; BMI, body mass index; VP, vaginal pressure; TVA, transversus abdominis muscle.

Supplementary Table S2. Within-session, within-examiner repeatability of transversus abdominis muscle thickness measurements.

Measurement condition	n	Single-measure ICC, ICC(A,1) (95% CI)	Average-measure ICC, ICC(A,3) (95% CI)
At rest	213	0.89 (0.86–0.92)	0.96 (0.95–0.97)
Maximal inspiration	213	0.86 (0.82–0.89)	0.95 (0.93–0.96)
Maximal expiration	213	0.91 (0.89–0.93)	0.97 (0.96–0.98)
Abdominal drawing-in maneuver	213	0.87 (0.84–0.90)	0.95 (0.94–0.96)

ICCs were calculated using a two-way mixed-effects model with an absolute-agreement definition. ICC(A,1) represents the repeatability of a single measurement, whereas ICC(A,3) represents the repeatability of the mean of three measurements. ICC values of <0.50, 0.50–0.75, 0.75–0.90, and >0.90 were interpreted as poor, moderate, good, and excellent reliability, respectively.

CI, confidence interval; ICC, intraclass correlation coefficient; TVA, transversus abdominis.

Supplementary Table S3. Extent and patterns of missing data among the 263 clinically eligible participants.

Panel A. Variable-specific missingness.

Variable	Available, n	Missing, n	Missing, %
Age	263	0	0.0
BMI	263	0	0.0
History of childbirth	263	0	0.0
Number of deliveries	263	0	0.0
Postmenopausal status	263	0	0.0
Hypertension	263	0	0.0
Diabetes mellitus	263	0	0.0
Chronic kidney disease	263	0	0.0
Hyperlipidemia	263	0	0.0
OABSS Q1	253	10	3.8
OABSS Q2	255	8	3.0
OABSS Q3	242	21	8.0
OABSS Q4	231	32	12.2
Total OABSS	220	43	16.3
Voided volume	263	0	0.0
Maximum flow rate	263	0	0.0
Postvoid residual urine	263	0	0.0
Duration of sustained PFM contraction	259	4	1.5
Maximal PFM contraction pressure	259	4	1.5
TVA thickness at rest	260	3	1.1
TVA thickness during maximal inspiration	260	3	1.1
TVA thickness during maximal expiration	260	3	1.1
TVA thickness during the abdominal drawing-in maneuver	260	3	1.1
Absolute TVA thickening	260	3	1.1
TVA percentage thickness change	260	3	1.1

Percentages were calculated using all 263 clinically eligible participants as the denominator. The total OABSS was considered available only when all four OABSS items had been completed. Item-level missingness could overlap within participants. BMI, body mass index; OABSS, Overactive Bladder Symptom Score; PFM, pelvic floor muscle; TVA, transversus abdominis muscle.

Panel B. Comparison according to OABSS total score availability.

Variable	Total available OABSS (n=220)	Total unavailable OABSS (n=43)	Absolute standardized difference	P value
Age, years	67.0 [59.0–74.0]	68.0 [61.0–75.0]	0.11	0.421
BMI, kg/m²	22.8 [20.6–25.2]	23.0 [20.7–25.5]	0.06	0.684
History of childbirth, n (%)	211 (95.9)	41 (95.3)	0.03	0.698
Number of deliveries	2.0 [2.0–3.0]	2.0 [2.0–3.0]	0.05	0.703
Postmenopausal status, n (%)	203 (92.3)	39 (90.7)	0.06	0.758
Hypertension, n (%)	102 (46.4)	21 (48.8)	0.05	0.767
Diabetes mellitus, n (%)	59 (26.8)	12 (27.9)	0.02	0.882
Chronic kidney disease, n (%)	59 (26.8)	13 (30.2)	0.08	0.646
Hyperlipidemia, n (%)	60 (27.3)	11 (25.6)	0.04	0.819
Voided volume, mL	198.0 [156.0–275.0]	193.0 [150.0–266.0]	0.09	0.548
Maximum flow rate, mL/s	21.2 [18.4–26.4]	20.8 [17.9–25.8]	0.10	0.477
Postvoid residual urine, mL	31.0 [16.0–46.0]	33.0 [18.0–49.0]	0.12	0.392
Duration of sustained PFM contraction, s	12.0 [6.0–15.0]	12.0 [6.0–15.0]	0.04	0.812
Maximal PFM contraction pressure, cmH_2O	21.0 [14.0–32.0]	20.0 [14.0–31.0]	0.10	0.462
TVA thickness during the abdominal drawing-in maneuver, mm	4.5 [3.9–5.7]	4.4 [3.8–5.6]	0.08	0.598

Data are presented as median [interquartile range] or n (%). Groups were compared using the Mann–Whitney U test and Pearson's chi-squared or Fisher's exact test, as appropriate. Absolute standardized differences were also calculated. PFM data were available for 216 and 43 participants, and TVA data for 217 and 43 participants, in the OABSS total score available and unavailable groups, respectively. BMI, body mass index; OABSS, Overactive Bladder Symptom Score; PFM, pelvic floor muscle; TVA, transversus abdominis muscle.

Supplementary Table S4. Sensitivity analysis using multiple imputation for factors associated with overactive bladder among the 263 clinically eligible participants.

Variable	Multiple-imputation analysis, adjusted OR (95% CI), n=263	P-value
Age, per 1-year increase	1.012 (0.971–1.056)	0.545
BMI, per 1 kg/m^2 increase	1.095 (0.995–1.206)	0.060
Number of deliveries, per 1-delivery increase	1.079 (0.763–1.527)	0.670
Postmenopausal status, yes vs. no	0.481 (0.114–2.017)	0.317
Hypertension, yes vs. no	4.523 (2.297–8.906)	<0.001
Maximal PFM contraction pressure, per 5-cmH_2O increase	0.716 (0.603–0.851)	<0.001
TVA thickness during the abdominal drawing-in maneuver, per 1 mm increase	0.526 (0.382–0.724)	<0.001

The primary complete-case multivariable analysis is presented in Table 6 of the main manuscript and is not repeated here. The multiple-imputation sensitivity analysis included all 263 clinically eligible participants. Missing data were handled using 50 imputed datasets under a missing-at-random assumption. Missing OABSS item scores (Q1–Q4) were imputed using ordinal logistic regression, whereas missing maximal PFM contraction pressure and TVA thickness during the abdominal drawing-in maneuver were imputed using predictive mean matching. In each imputed dataset, total OABSS was calculated from the four imputed item scores, and OAB status was passively derived according to the prespecified definition of a total OABSS ≥3 with an urgency score (Q3) ≥2; binary OAB status was not imputed separately. The primary multivariable logistic regression model was fitted separately in each imputed dataset, and the resulting estimates and standard errors were combined using Rubin’s rules. ORs for maximal PFM contraction pressure and TVA thickness are expressed per 5-cmH_2O increase and per 1 mm increase, respectively; ORs < 1 indicate lower odds of OAB with higher values. OR, odds ratio; CI, confidence interval; OAB, overactive bladder; OABSS, Overactive Bladder Symptom Score; PFM, pelvic floor muscle; TVA, transversus abdominis muscle.
